# Association of maternal and cord vitamin B12 levels with anthropometry in term neonates born to malnourished mothers in coastal South India

**DOI:** 10.12688/f1000research.150696.1

**Published:** 2024-05-24

**Authors:** Sugapradha GR, Ramesh Holla, Poornima Manjrekar, Suchetha Rao

**Affiliations:** 1 Pediatrics, Kastrurba Medical College Mangalore Manipal Academy of Higher Education, Manipal, Karnataka, 576104, India; 2 Community Medicine, Kasturba Medical College Mangalore Manipal Academy Of Higher Education, Manipal, Karnataka, 576104, India; 3 Biochemistry, Kasturba Medical College Mangalore Manipal Academy Of Higher Education, Manipal, Karnataka, 576104, India

**Keywords:** Health, low birth weight, malnourished, neonate, pregnant women, umbilical cord, vitamin B12

## Abstract

**Background:**

Malnourished pregnant women are at increased risk of micronutrient deficiency. We assessed the vitamin B12 status in both malnourished and normally nourished pregnant women and their neonates. Additionally, we studied the association between maternal B12 levels, cord B12 levels and neonatal anthropometry.

**Methods:**

This cross-sectional study enrolled 63 malnourished and 63 normally nourished mothers and neonates. Maternal and cord blood samples were collected at the time of delivery for estimation of vitamin B12 levels. Maternal and cord vitamin B12 levels were compared using the Mann–Whitney U test. Neonatal anthropometry was correlated with maternal and cord B12 levels using Spearman’s correlation. Data were analyzed using SPSS version 25.

**Results:**

Mean maternal age was 26.58 yrs. The median cord B12 levels were lower than the maternal B12 levels. Maternal B12 levels showed a strong positive correlation with cord B12 levels (rho = 0.879; p < 0.001). Maternal (p < 0.001) and cord (p < 0.001) vitamin B12 levels were significantly lower in the malnourished group than in the normally nourished group. In malnourished group, 66.8% mothers and 95.2% neonates were Vitamin B12 deficient, whereas 1.5% mothers and 4.7% neonates were vitamin B12 deficient in normally nourished group. In the malnourished group, maternal B12 levels were positively correlated with birth weight (rho 0.363, p = 0.003) and length (rho 0.330, p =0.008), whereas cord B12 levels were positively correlated with birth weight in the normally nourished group. (rho 0.277 p= 0.028)

**Conclusion:**

High rates of vitamin B12 deficiency were observed in malnourished mothers and neonates. There was a positive correlation between birth weight, length, and maternal vitamin B12 levels in malnourished mothers. These findings emphasize the need to address maternal malnutrition and vitamin B12 deficiency to improve neonatal health.

## Introduction

Vitamin B12, also known as cobalamin, is a micronutrient essential for cell growth and differentiation. ^1^ This essential vitamin plays a crucial role in various biochemical processes, particularly in the synthesis of DNA, in conjunction with folic acid. ^2^ Vitamin B12 is commonly found in animal-based food products such as dairy, fish, eggs, and poultry. Consuming these foods is an effective way to acquire vitamin B12 from the diet. While the vegetarian population is at a heightened risk of deficiency, it is also prevalent among non-vegetarians. ^3^

Vitamin B12 deficiency is a major health concern worldwide. Pregnant women and young children are at an increased risk of facing this deficiency. ^4^ Regional disparities in the occurrence of vitamin B12 deficiency throughout pregnancy have been observed, indicating varying levels of risks across different regions. ^5^

Inadequacy of vitamin B12 in pregnancy has been linked to unfavourable pregnancy outcomes, including spontaneous abortions, stillbirth, neural tube defects, intrauterine growth restriction, low birth weight, and premature delivery. ^6^^–^^10^ Deficiency of vitamin B12 is also implicated in increased risk of gestational diabetes mellitus. ^11^ Adequate levels of vitamin B12 during early childhood promote growth and prevent cognitive impairment. A systematic review by Rogne et al. reported no clear linear relationship between birth weight and maternal vitamin B12 levels during pregnancy. However, deficiency was linked to a higher number of neonates born with low birth weight. ^12^

Vitamin B12 deficient mothers may produce breast milk with insufficient levels of vitamin B12, and their exclusively breast-fed infants may develop symptoms in the postnatal period. These infants may have apathy, anorexia, irritability, failure to thrive, delayed or regression of milestones, pancytopenia, hypotonia, and seizures. Early symptoms may be nonspecific and pose challenges for identification. Vitamin B12 levels in expectant mothers are thought to influence the vitamin B12 status of the fetus and infant. The association between maternal, neonatal, and infant vitamin B12 levels has been heterogenous. ^13^^–^^17^

Pregnant women experiencing malnutrition face a heightened risk of micronutrient deficiencies, pregnancy complications, and perinatal outcomes. ^18^ There are limited data regarding the occurrence of vitamin B12 deficiency in malnourished expectant mothers and its impact on neonatal outcomes.

We aimed to assess the vitamin B12 status in malnourished and normally nourished pregnant women and to study the association between maternal B12 levels and cord B12 levels and neonatal anthropometry.

## Methods

### Study setting and period

This cross-sectional study was conducted in a community maternity facility affiliated with the Kasturba Medical College, Mangalore, Manipal Academy of Higher Education, Manipal, India. The study was conducted between August 2020 and July 2021.

### Study design

This cross-sectional study followed the standard Strengthening Reporting of Observational Studies in Epidemiology (STROBE) statement guidelines. ^19^ The reporting guidelines contain the completed STROBE checklist. ^20^

### Study participants

The first group consisted of malnourished mothers and their neonates. The other group was comprised of normally nourished mothers and their neonates.

### Inclusion criteria

All term neonates born to normal and malnourished mothers under singleton pregnancies.

### Exclusion criteria

(a) Pregnant women <18 years and > 35 years.
(b) Pregnancy-induced hypertension/preeclampsia.
(c) Maternal history of smoking and substance abuse.
(d) Women with chorionic disease.
(e) Women with diabetes mellitus.
(f) Women with pre-existing cardiovascular hypertension or renal disorders.
(g) Pregnant women receiving multivitamin supplements other than routine iron and folic acid.
(h) Severe anemia.
(i) Blood transfusions within 3 months.
(j) Neonates with major congenital anomalies.

### Sample size and technique

The sample size was determined based on a prior study by Finkelstein JL, et al., which reported the extent of vitamin B12 deficiency in South Indian pregnant women was 51%. ^21^ With 95% confidence interval, 80% power, and a 5% level of significance. The sample size was calculated to be 63 for each group. A random sampling technique was used to reduce selection bias.

### Operational definition

Malnourished mother: A mother with a pre-pregnancy weight of less than 45 kg documented in antenatal records. ^22^

Small for gestational age (SGA) is defined as birth weight < 10 ^th^ percentile for gestation. ^23^

Vitamin B12 level ^24^

       Normal      >300 pg/ml

       Insufficiency   200-300 pg/ml

       Deficiency    <200 pg/ml

### Ethics and data collection

Our research adhered to the ethical principles of world medical association Helsinki declaration of 1964 and later amendments. The study was carried out after obtaining permission from the Institutional Ethical Committee of KMC Mangalore, Manipal Academy of Higher Education (approval number IEC KMC MLR-08/2020/249 letter dated 20/08/2020), and necessary permissions were sought from hospital authorities. Based on the inclusion and exclusion criteria, suitable subjects were approached and provided with a participant information sheet. The purpose of the study was explained in their native language, and consent was obtained from those willing to participate. After obtaining consent, the participants were recruited for the study. Participation in the study was voluntary, and the complete anonymity of the research participants was maintained. We ensured confidentiality of the collected data.

Maternal and neonatal details were obtained from antenatal and hospital records. Neonatal anthropometric measurements were performed by an investigator. Weight was measured to the nearest 0.01 kg using an electronic weighing machine, while the length of the newborn was estimated using an infantometer to the nearest to 0.1 cm, and the circumference of the head was calculated using a non-stretchable tape nearest to 0.1 cm. Based on birth weight and gestational age, the babies were categorized as Large, Small, and Appropriate for Gestational Age. Maternal and neonatal details were documented using a semi-structured pretested pro forma.

### Sample collection

At the time of delivery, approximately 4 ml of maternal venous blood was collected in plain glass tubes, and 4 ml of blood from the umbilical cord was directly obtained via venipuncture of the umbilical vein. The specimens were then immediately placed on ice. Centrifugation was performed at a rate of 4000 revolutions per minute for 5 min. Subsequently, the plasma was separated and stored at -80°C pending analysis.

### Test procedure

Serum vitamin B12 levels were measured using a vitamin B12 ELISA kit (Epitope Diagnostics, San Diego, CA 92121, USA) at the Department of Biochemistry, KMC Centre for Basic Sciences, Bejai, Mangalore. In this assay, antibodies specific to vitamin B12 were bound to the surface of a microtiter plate. Standards or samples containing vitamin B12 and conjugated vitamin B12-peroxidase were added to the wells of a microtiter plate. Free vitamin B12 and enzyme labelled vitamin B12 compete for the binding sites on the antibodies. Following one hour of incubation at room temperature, the wells were washed with a diluted washing solution to eliminate any unbound materials. Subsequently, the substrate solution was added and incubated for 20 min, leading to the development of a blue color. This color development was inhibited by adding a stop solution, after which it turned yellow. Color intensity was measured photometrically at 450 nm. The concentration of vitamin B12 displayed an inverse relationship with the color intensity observed in the test sample.

### Outcome variables

Percentage of maternal and cord B12 levels in normal, insufficient and deficient category.

Relation between maternal and cord B12 level and neonatal anthropometry.

### Statistical analysis

The collected data were entered into the Statistical Package for IBM (SPSS) Statistics for Windows version 25.0. Armonk, NY: IBM Corp. for analysis. Anthropometric and demographic data were expressed as means and standard deviations or proportions. Vitamin B12 levels are expressed as medians and interquartile ranges. Maternal and neonatal characteristics were compared in both groups using the independent sample t-test and chi-square test. Vitamin B12 levels were compared between groups using the Mann–Whitney U test. Correlations between maternal and cord B12 levels and neonatal anthropometries were assessed using the Spearman correlation test.

## Results

Of the 126 pregnant women enrolled, 63 were malnourished and 63 were normal nourished. The mean age of the study subject was 26.58 (± 3.79) years. The mean pre-pregnancy weight was 48.68 (± 6.17) kg. Among these women, 75 (59.5%) were primigravidas. mixed diet was followed by 117 (92.9%) women, and all women consumed milk. Among the neonates, 64 (50.8%) were male and 62 (49.2) were female. The mean gestational age of the neonates was 38.39 (± 1.19) weeks. Mean birth weight was 2.77 (± 0.35) kg and 22 (17.5%) neonates belonged to small for gestation age (SGA) category. Maternal and neonatal characteristics are shown in Table 1.

**Table 1.** Maternal and neonatal characteristics.

Parameter	mean (SD) or %
**Maternal characteristics**	
Age, yrs	26.58 (3.79)
Height, cm	157.83 (4.20)
Pre-pregnancy weight, kg	48.68 (6.17)
Maternal weight at delivery, kg	56.96 (6.34)
BMI kg/m ^2^	19.35 (2.79)
Weight gain during pregnancy, kg	8.30 (0.85)
Gravida * (%)	
Primigravida	75 (59.5)
Multigravida	51 (40.5)
Diet * (%)	
Vegetarian	9 (7.1)
Mixed diet	117 (92.9)
Milk consumption * (%)	
Yes	126 (100)
No	0 (0)
**Neonatal characteristics**	
Gestational age (weeks)	38.39 (1.19)
Birth weight (kg)	2.77 (0.35)
Gestational weight category * (%)	
Appropriate for Gestational Age	104 (82.5)
Small for Gestational Age	22 (17.5)
Length (cm)	48.87 (0.99)
Head circumference (cm)	33.94 (0.97)
Gender * (%)	
Female	62 (49.2)
Male	64 (50.8)

SD standard deviation.

* Values expressed as %.

Birth weight, length, and head circumference of neonates born to malnourished mothers were lower than those of neonates born to mothers with normal nourishment. Sixteen (25.4%) neonates of malnourished mother group were SGA. A comparison of the anthropometric parameters of neonates born to malnourished and normal-nourished mothers is shown in Table 2.

**Table 2.** Neonatal characteristics among normally nourished and malnourished mothers.

Parameter	Neonates of normal nourished Mothers Mean (SD)	Neonates of malnourished Mothers Mean (SD)	P-value
Gender * (%)			
Female	29 (46)	33 (52.4)	0.476
Male	34 (54)	30 (47.6)	
Gestational age, weeks	38.32 (1.11)	38.46 (1.28)	0.504
Birth weight, kg	2.95 (0.38)	2.57 (0.19)	<0.001
Birth weight category * (%)			
AGA	57 (90.5)	47 (74.6)	0.033
SGA	6 (9.5)	16 (25.4)	
Length, cm	49.48 (0.72)	48.25 (0.84)	<0.001
Head circumference, cm	34.19 (1.12)	33.68 (0.71)	<0.001

* Values expressed as %.

The median cord B12 value was considerably lower than the maternal B12 value, as shown in Table 3. Both maternal and cord B12 levels were significantly lower in the malnourished group than in the normal-nourished group ( Table 4). In malnourished group, 66.8% mothers and 95.2% neonates were vitamin B12 deficient. In normal nourished group, only 1.5% mothers and 4.7% neonates were deficient. The maternal and cord B12 levels in both groups are shown in Table 5. In the malnourished group, the maternal B12 level showed a positive correlation with birth weight (rho 0.363, p = 0.003) and length (rho 0.330, p = 0.008), whereas in the normally nourished group, the cord B12 level was positively correlated with birth weight (rho 0.277 p = 0.028) ( Tables 6 and 7), respectively.

**Table 3.** Maternal and cord vitamin B12 levels.

Vitamin B12 levels (pg/ml)	Median (Interquartile range)
Cord	191.5 (89.63-358.75)
Maternal	257.9 (167-505)

**Table 4.** Maternal and cord vitamin B12 levels in malnourished and normal nourished mothers.

Vitamin B12 Levels (pg/ml)	Normal nourished mothers Median (IQR)	Malnourished mothers Median (IQR)	P value
Maternal	499 (357-748)	168 (123-208)	<0.001
Cord	352 (259.60-438)	93 (57.9-119)	<0.001

**Table 5.** Maternal and cord vitamin B12 level categories in normally nourished and malnourished mothers.

Categories	Normally nourished n (%)	Malnourished n (%)	P-value
Maternal Vitamin B12			
Normal	56 (89)	3 (4.7)	<0.001
Insufficient	6 (9.5)	18 (28.5)	
Deficient	1 (1.5)	42 (66.8)	
Cord Vitamin B12			
Normal	43 (68.3)	2 (3.2)	**<0.001**
Insufficient	17 (27)	1 (1.6)	
Deficient	3 (4.7)	60 (95.2)	

**Table 6.** Correlation of maternal, cord vitamin B12 and neonatal anthropometry in normal nourished women.

Parameter	Maternal Vitamin B12		Cord Vitamin B12	
	rho	P value	rho	P value
Birth weight	0.228	0.073	0.277	0.028
Length	0.064	0.621	0.007	0.956
Head circumference	0.049	0.703	-0.021	0.869

**Table 7.** Correlation of maternal, cord vitamin B12 and neonatal anthropometry in malnourished women.

Parameter	Maternal vitamin B12		Cord vitamin B12	
	rho	P value	rho	P value
Birth weight	0.363	0.003	0.133	0.298
Length	0.330	0.008	0.011	0.934
Head circumference	0.09	0.483	0.166	0.193

## Discussion

During gestation, the fetus depends entirely on maternal nutrition. Low levels of vitamin B12, primarily sourced from the mother at certain stages of gestation, can lead to adverse outcomes, such as low birth weight and early postnatal effects on cognitive development. ^25^^–^^27^

Vitamin B12 deficiency is widespread globally and affects various populations. A study by Ramírez-Vélez et al in a sample representative of Columbian pregnant women, found that 18.6% of the population were deficient in Vitamin B12 and 41.3% exhibited insufficiency. ^28^

In a nutritional intervention study that enrolled moderately malnourished Malawian mothers, 20.9% of the participants were deficient in Vitamin B12. ^29^

There is a significant occurrence of Vitamin B12 deficiency in Indian women during pregnancy. Studies from Western and Northern parts of India have reported highest occurrence of Vitamin B12 deficiency during pregnancy, ranging from 70-74%. ^5^

A community based cross sectional study by Barney et al., enrolling pregnant women in rural South India, reported an extent of 55% Vitamin B12 deficiency. They found obesity and being in the first trimester of pregnancy to be independent risk factors. ^30^

A study conducted by Katre et al. in Pune, which recruited pregnant women during 17 weeks of gestation, indicated that approximately 80% of rural and 65% of urban women exhibited low Vitamin B12 status. ^31^

A prospective cohort study involving pregnant women in the age group of 18-45 years from urban hospitals of Bangalore reported low plasma vitamin B12 levels in 48.6% of women. ^32^ In the present study, 66.8% women and 95.2% neonates of malnourished group exhibited vitamin B12 deficiency.

A review by Sukumaran et al. suggested that even within non-vegetarian populations, vitamin B12 insufficiency during pregnancy is frequently observed. ^33^ Similar results were observed in our study.

Concentrations of vitamin B12 showed a declining trend from the first trimester to the third trimester, as reported by Sukumaran et al. ^33^ Similar observations were reported by Sobowale et al. from Bangladesh. ^34^ A cohort study involving healthy adolescent pregnant women from New York observed declining levels of maternal serum cobalamin levels from mid gestation to delivery. They found that maternal vitamin B12 concentrations at delivery were associated with infants’ vitamin B12 concentrations. ^35^

A study by Adaikalakoteswari et al. found that offspring of mothers with low B12 levels exhibited significantly lower levels of vitamin B12 compared to those born to mothers with normal levels. ^17^

A Study by Finkelstein e t al., which recruited pregnant women around 12 weeks gestation from South India, observed that vitamin B12 deficiency prevalent in around 63% women during early pregnancy. ^35^ This deficiency served as predictor of neonatal vitamin B12 status. Specifically, lower maternal serum vitamin B12 levels are associated with a two-fold increased risk of neonatal vitamin B12 deficiency at birth. ^36^

Yildizdas et al. conducted a study involving Turkish mothers and neonates, revealing alarmingly high rates of vitamin B12 deficiency, reaching 96.3%). They noted that micronutrient levels in the cord blood surpassed maternal levels. There was a negative correlation between birth weight, head circumference, and cord blood Cbl levels. Interestingly, maternal Cbl levels did not show any correlation with neonatal anthropometry. ^37^ In the present study, cord B12 levels were lower than the maternal levels, and cord levels showed a positive correlation with maternal levels.

In a retrospective study by Yuan et al. in Eastern China involving 11,549 pregnant women, it was found that elevated maternal serum Vitamin B12 levels correlated with higher birth weight and an increased risk of newborn birth being large. ^7^

Sukumar et al. concluded that there is no consistent relationship between vitamin B12 sufficiency and LBW. ^33^ MAASTHI birth cohort study by Deepa et al. in South India found no association between vitamin B12 status and birth weight. ^32^

Hay et al. reported a negative association between cord Cbl and birth weight. The heaviest babies had lower cord Cbl levels. A similar negative association was observed between birth length and head circumference also. ^38^

A secondary analysis of 709 Canadian mother newborn dyads by Tan et al. observed that maternal serum Vitamin B12 biomarkers did not show any association with birth weight or head circumference. ^39^ In the present study, maternal B12 levels showed a positive correlation with birth weight (rho 0.363, p = 0.003) and length (rho 0.330, p = 0.008) but not with head circumference. In the normally nourished group, cord B12 levels showed a positive correlation with birth weight only (rho 0.277, p = 0.028).

Limitations: This study was conducted in a tertiary care hospital, which may restrict the generalizability of its findings to a broader community. Maternal vitamin B12 levels measured only at the time of delivery may not capture fluctuations throughout pregnancy. A smaller sample size raises concerns regarding the applicability of the results. The presence of coexisting micronutrient deficiencies in malnourished women poses a risk of confounding bias in the interpretation of results.

In conclusion, the present study revealed high rates of vitamin B12 deficiency in malnourished mothers and their neonates. Additionally, There was a positive correlation between birth weight, length, and maternal vitamin B12 levels in malnourished mothers. These findings emphasize the need to address maternal malnutrition and vitamin B12 deficiency to improve neonatal health.

### Ethical considerations and consent

Institutional ethical approval was obtained from the ethics committee of Kasturba Medical College Mangalore (IEC KMC MLR-08/2020/249). Permission from the medical superintendent of the hospital was obtained prior to the study. A patient information sheet was provided and the purpose of the study was explained to the parents. Written informed consent was obtained from parents or legal guardians following the Helsinki Declaration. Participation in the study was voluntary, and the complete anonymity of the research participants was maintained. We ensured confidentiality of the collected data.

## Data Availability

Association of maternal and cord vitamin B12 levels with anthropometry in term neonates born to malnourished mothers in Coastal South India. figshare. Dataset. https://doi.org/10.6084/m9.figshare.25622415. ^20^ Figshare: STROBE checklist for ‘Association of maternal and cord vitamin B12 levels with anthropometry in term neonates born to malnourished mothers in Coastal South India.’ https://doi.org/10.6084/m9.figshare.25622415. ^20^ The data are available under the terms of the Creative Commons Attribution 4.0 International license (CC-BY 4.0).
