# Supplementary material for: Association of maternal and cord vitamin B12 levels with anthropometry in term neonates born to malnourished mothers in coastal South India
Source: F1000Res. 2024 Jul 23;13:530. Originally published 2024 May 24. [Version 2] doi: 10.12688/f1000research.150696.2 (PMC11299053; doi:10.12688/f1000research.150696.2)
Supplement: Peer review history

# Reviewer report: Reviewer response for version 2

**DOI:** 10.5256/f1000research.169306.r306042

**Published:** 2024-08-02

**Authors:** Noor Rohmah Mayasari

**Affiliations:** 1 Nutrition, Universitas Negeri Surabaya, Surabaya, East Java, Indonesia

No further comments

Is the work clearly and accurately presented and does it cite the current literature?

Partly

If applicable, is the statistical analysis and its interpretation appropriate?

Partly

Are all the source data underlying the results available to ensure full reproducibility?

Yes

Is the study design appropriate and is the work technically sound?

Yes

Are the conclusions drawn adequately supported by the results?

Partly

Are sufficient details of methods and analysis provided to allow replication by others?

Yes

Reviewer Expertise:

Nutritional epidemiology, anemia, food security

I confirm that I have read this submission and believe that I have an appropriate level of expertise to confirm that it is of an acceptable scientific standard.

# Reviewer report: Reviewer response for version 2

**DOI:** 10.5256/f1000research.169306.r306041

**Published:** 2024-08-01

**Authors:** Rathika Damodara Shenoy

**Affiliations:** 1 American University of Antigua, Antigua, USA

The author in the revised version has addressed my queries. No further comments.

Is the work clearly and accurately presented and does it cite the current literature?

Yes

If applicable, is the statistical analysis and its interpretation appropriate?

Yes

Are all the source data underlying the results available to ensure full reproducibility?

Yes

Is the study design appropriate and is the work technically sound?

Yes

Are the conclusions drawn adequately supported by the results?

Yes

Are sufficient details of methods and analysis provided to allow replication by others?

Partly

Reviewer Expertise:

Perinatology, Metabolic disorders, Dysmorphology

I confirm that I have read this submission and believe that I have an appropriate level of expertise to confirm that it is of an acceptable scientific standard.

# Reviewer report: Reviewer response for version 1

**DOI:** 10.5256/f1000research.165288.r289090

**Published:** 2024-06-29

**Authors:** Noor Rohmah Mayasari

**Affiliations:** 1 Nutrition, Universitas Negeri Surabaya, Surabaya, East Java, Indonesia

General comments:

 The study aimed to assess vitamin B12 status in malnourished and normally nourished pregnant women and study the association between maternal B12 level and cord B12 labels and neonatal anthropometry. However, the paper hasn't discussed the findings related to the aim.

 Introduction:

1. Please modify the introduction structure (the paragraph doesn't have the main sentence of each). Some paragraphs are too short (less than three sentences)

Methods:

1. Please clarify the study design, is there a case-control or cross-sectional study?
2. Why exclude severe anaemia, is there any specific reason?
3. Malnourished is define as a pre-pregnancy body weight less than 45kg. Why use body weight rather than pre-pregnancy body mass index?

Results:

1. Explain the reason why in the table presentation authors use different display outcomes (table 2 (mean and SD), table 3 (median and interquartile range).
2. Table 2 expresses as (%) or (mean and SD)?
3. Tables 2-5can be merged as 1 table and Tables 6-7 can be merged as 1 table.
4. From the study authors can analyze RR or OR

Discussion:

1. What Is the key finding of this study?
2. Please modify the discussion structure (the paragraph doesn't have the main sentence of each). Some paragraphs are too short (less than three sentences).
3. The data shows that maternal vitamin B12 positively correlated with Birth weight and length among malnourished women. However, this correlation is not significant with cord vitamin B12, what is that mean and implication?
4. Are there any maternal characteristics that effect the main outcome as confounding factors?

Is the work clearly and accurately presented and does it cite the current literature?

Partly

If applicable, is the statistical analysis and its interpretation appropriate?

Partly

Are all the source data underlying the results available to ensure full reproducibility?

Yes

Is the study design appropriate and is the work technically sound?

Yes

Are the conclusions drawn adequately supported by the results?

Partly

Are sufficient details of methods and analysis provided to allow replication by others?

Yes

Reviewer Expertise:

Nutritional epidemiology, anemia, food security

I confirm that I have read this submission and believe that I have an appropriate level of expertise to confirm that it is of an acceptable scientific standard, however I have significant reservations, as outlined above.

# Response

**Published:** 2024-07-17

**Authors:** SUCHETHA S RAO

**Affiliations:** Pediatrics, Kasturba Medical College Mangalore, Mangalore, Karnataka, India

***Reviewer Comment:***

**General comments:**

 The study aimed to assess vitamin B12 status in malnourished and normally nourished pregnant women and study the association between maternal B12 level and cord B12 labels and neonatal anthropometry. However, the paper hasn't discussed the findings related to the aim.

***------------------***

***Author reply :*** Thank you for reviewing our article and giving valuable suggestions. We have  modified the article as per your suggestions. We have added additional data related to aim.

***------------------***

***Reviewer Comment:***

**Introduction:**

 1.Please modify the introduction structure (the paragraph doesn't have the main sentence of

    each). Some paragraphs are too short (less than three sentences)

***------------------***

***Author reply :*** Introduction has been modified as per the suggestion

    First paragraph- Role of Vitamin B12 in health , sources

    Second paragraph- Deficiency and health burden , prevalence and regional disparities of deficiency

    Third paragraph -Vitamin B12 deficiency adverse outcomes in pregnancy , and effect of maternal deficiency on child health

     Fourth paragraph - Need for the present study and aims of the study

***------------------***

***Reviewer Comment:***

**Methods**

 1. Please clarify the study design, is there a case-control or cross-sectional study?

     Author reply     Cross sectional study

 2. Why exclude severe anaemia, is there any specific reason?

    Author reply    Severe anemia itself can lead to low birth weight babies.  

 3 .Malnourished is define as a pre-pregnancy body weight less than 45kg. Why use body weight rather

    than pre-pregnancy body mass index?

***------------------***

***Author reply :***  Antenatal records had pre pregnancy weight documented not BMI ,hence we chose weight criteria for defining malnourished mother

***------------------***

***Reviewer Comment:***

**Results:**

 1.Explain the reason why in the table presentation authors use different display outcomes (table 2 (mean and SD), table 3 (median and interquartile range).

***------------------***

***Author reply :***

    Table 2 mentions about anthropometric measures which followed normal distribution , hence we used mean and SD

    Table 3 mentions about vitamin B12 levels which did not follow normal distribution , hence used Median and Interquartile range

***------------------***

***Reviewer Comment:***

 2.Table 2 expresses as (%) or (mean and SD)?

***------------------***

***Author reply :***

     Expressed as Mean and SD or as (%). values marked * are expressed as (%)

***------------------***

***Reviewer Comment:***

 3.Tables 2-5 can be merged as 1 table and Tables 6-7 can be merged as 1 table.

***------------------***

***Author reply :***

     We have merged table 4 and 5 as one table ( new table no 4 )

     We have merged table 6 and 7 as one table ( new table no 5 )

***------------------***

***Reviewer Comment:***

 4.From the study authors can analyze RR or OR

***------------------***

***Author reply :***

    We have calculated OR. OR for malnourished mothers to have vitamin B12 deficiency is 160 ( 95%  CI   39.43,  649.27)

***------------------***

***Reviewer Comment:***

**Discussion:**

 1.What Is the key finding of this study?

***------------------***

***Author reply :***

1. A significant proportion, about 53%, of women in the study population have insufficient or deficient B12 status despite a mixed diet
2. The median cord B12 value was considerably lower than the maternal B12 value and there was a strong positive correlation between maternal and cord B12 values
3.  Both maternal and cord B12 levels were significantly lower in the malnourished group than in the normal-nourished group
4. In malnourished group , maternal vitamin B12 positively correlated with Birth weight and length. Cord B12 showed no correlation with neonatal anthropometry

***------------------***

***Reviewer Comment:***

 2.  Please modify the discussion structure (the paragraph doesn't have the main sentence of

      each). Some paragraphs are too short (less than three sentences).

***------------------***

***Author reply :***

 We have modified the discussion as per the suggestion

***------------------***

***Reviewer Comment:***

 3.  The data shows that maternal vitamin B12 positively correlated with Birth weight and length among malnourished women. However, this correlation is not significant with cord vitamin B12, what is that mean and implication?

***------------------***

***Author reply :***

      Maternal Vitamin B12 levels during pregnancy may directly influence growth of fetus , leading to higher birth weight and increased length. This implies that adequate maternal Vitamin B12 level is crucial for fetal growth. Low levels of maternal vitamin B12, at certain stages of  gestation, can lead to adverse outcomes, such as  low birth weight and shorter  neonates. Previous studies have reported declining concentrations of maternal vitamin B12 from  first trimester to the third trimester. Cord blood Vitamin B12 levels reflect status of Vitamin B12 of neonate at birth. Absence of correlation with cord Vitamin B12 means that Cord Vitamin B12 may not be a direct measure of ongoing maternal B12 influence during pregnancy. Critical periods of Vitamin B12 influence may occur at certain stages of gestation which may not be captured by Cord blood B12 value. This implies the need to maintain adequate maternal Vitamin B12 levels throughout pregnancy for better neonatal outcome.

***------------------***

***Reviewer Comment:***

 4.   Are there any maternal characteristics that effect the main outcome as confounding factors?

***------------------***

***Author reply :*** Co existing micronutrient deficiencies in malnourished mothers  may  effect the main outcome as  confounding factors. This has been mentioned in limitations.

***------------------***

# Reviewer report: Reviewer response for version 1

**DOI:** 10.5256/f1000research.165288.r289097

**Published:** 2024-06-22

**Authors:** Rathika Damodara Shenoy

**Affiliations:** 1 American University of Antigua, Antigua, USA

The cross-sectional study compares the maternal and cord blood B12 levels in normally nourished and malnourished mothers at term. The authors report lower maternal and cord blood B12 values in the malnourished group and a strong positive correlation between the measures.  The correlations between B12 values and newborn anthropometry are weak to none.

 The results reveal that a significant proportion, about 53%, of women in the study population have insufficient or deficient B12 status despite a mixed diet. It may be worthwhile to compare the maternal demography of the two groups (like Table 2 on newborn data). The authors can investigate if maternal BMI and B12 correlation will provide additional information. The limitations are listed by the authors.

 The main takeaway from the study is the high prevalence of maternal B12 deficiency in a coastal fish-eating community. Food taboos targeting pregnant and postpartum women are widely prevalent in India and South Asian countries. ^1,2^ The authors should investigate qualitative and quantitative diet information, the coexistence of iron deficiency, and the clinical relevance in future studies with a larger sample size.

Is the work clearly and accurately presented and does it cite the current literature?

Yes

If applicable, is the statistical analysis and its interpretation appropriate?

Yes

Are all the source data underlying the results available to ensure full reproducibility?

Yes

Is the study design appropriate and is the work technically sound?

Yes

Are the conclusions drawn adequately supported by the results?

Yes

Are sufficient details of methods and analysis provided to allow replication by others?

Partly

Reviewer Expertise:

Perinatology, Metabolic disorders, Dysmorphology

I confirm that I have read this submission and believe that I have an appropriate level of expertise to confirm that it is of an acceptable scientific standard.

## References

[1] : Restricting diet for perceived health benefit: A mixed-methods exploration of peripartum food taboos in rural Cambodia. *Matern Child Nutr* .2023;19(3) : 10.1111/mcn.13517 e13517 10.1111/mcn.13517 37016926 PMC10262911

[2] : Prevalence of Thiamine Deficiency in Pregnancy and its impact on fetal outcome in an area endemic for thiamine deficiency. *PLoS Negl Trop Dis* .2023;17(5) : 10.1371/journal.pntd.0011324 e0011324 10.1371/journal.pntd.0011324 37253018 PMC10256141

# Response

**Published:** 2024-07-17

**Authors:** SUCHETHA S RAO

**Affiliations:** Pediatrics, Kasturba Medical College Mangalore, Mangalore, Karnataka, India

**Review comment : **The cross-sectional study compares the maternal and cord blood B12 levels in normally nourished and malnourished mothers at term. The authors report lower maternal and cord blood B12 values in the malnourished group and a strong positive correlation between the measures. The correlations between B12 values and newborn anthropometry are weak to none.

 The results reveal that a significant proportion, about 53%, of women in the study population have insufficient or deficient B12 status despite a mixed diet. It may be worthwhile to compare the maternal demography of the two groups (like Table 2 on newborn data).

**Author reply** : Thank you for reviewing our article and giving valuable suggestions. We have modified the article as per your suggestions. We have compared the maternal demography and details are added to table 2 along with newborn data

**Review comment : **The authors can investigate if maternal BMI and B12 correlation will provide additional information.

**Author reply : **Spearman correlation test for BMI and Vitamin B12 level was done. There was a positive correlation between the two measures. ( rho 0.590 , p <0.001) This observation has been added to result.

**Review comment** :The limitations are listed by the authors.

 The main takeaway from the study is the high prevalence of maternal B12 deficiency in a coastal fish-eating community. Food taboos targeting pregnant and postpartum women are widely prevalent in India and South Asian countries.1,2

**Author reply : **Thank you for highlighting the significance of food taboos for possible contribution towards maternal Vitamin B12 deficiency along with references. We have added this to discussion.

**Review comment **The authors should investigate qualitative and quantitative diet information, the coexistence of iron deficiency, and the clinical relevance in future studies with a larger sample size.

**Author reply **We will plan for future studies
